# Exploring the complex associations among risks of malnutrition, sarcopenia, and frailty in community-dwelling older adults

**DOI:** 10.1186/s11556-024-00354-7

**Published:** 2024-07-09

**Authors:** Halil Ibrahim Celik, Ferda Koc, Kübra Siyasal, Büsra Ay, Nazlı Bengu Ilter, Ozge Mengi Celik

**Affiliations:** 1 Bilge Çocuk Special Education and Rehabilitation Center, Beysukent, Çankaya, Ankara, s06800 Turkey; 2 Department of Nutrition and Dietetics, Faculty of Health Sciences, University of Health Sciences, Ankara, Turkey

**Keywords:** Sarcopenia, Frailty, Elderly, Mediator Effect, Moderator effect

## Abstract

**Background:**

Malnutrition, sarcopenia, and frailty are age-related conditions that are associated with multiple health-related negative outcomes. However, the complex associations between them remain to be elucidated. The aims of the study were to explore: (1) whether the risk of sarcopenia has a mediator effect on the association between risks of malnutrition and frailty; and (2) whether physical activity (PA) level modulates this mediator effect in community-dwelling older adults.

**Methods:**

This cross-sectional study involved 593 older adults (62.73% female; mean age = 71.35 ± 5.86 years). The Mini Nutritional Assessment-Short Form (MNA-SF), the SARC-F Questionnaire, and the FRAIL Questionnaire were used to assess the risks of malnutrition, sarcopenia, and frailty, respectively. The International Physical Activity Questionnaire Short Form (IPAQ-SF) was employed to assess PA level. Using the Hayes PROCESS macro (Models 4 and 7), mediation and moderated mediation analyses were performed.

**Results:**

The mediation analysis demonstrated that the MNA-SF had a significant effect on the SARC-F (B=-0.325; *p* < 0.001) and the SARC-F, in turn, had a significant effect on the FRAIL (B = 0.341; *p* < 0.001). The total (B=-0.171; *p* < 0.001), direct (B=-0.061; *p* = 0.001), and indirect (B=-0.111; bootstrap CI did not include zero, which indicates a significant effect) effects of MNA-SF on FRAIL were significant, showing that 65% of the association between the MNA-SF and FRAIL was explained by the SARC-F acting as a mediator. The moderated mediation analysis demonstrated that the association between MNA and SARC-F was moderated by the PA level (B = 0.253; *p* = 0.016). The SARC-F mediated and relatively enhanced the association between MNA-SF and FRAIL only in older adults with a moderate PA level (B=-0.120; CI: -0.154 to -0.085).

**Conclusions:**

The SARC-F partially mediates the association between the MNA-SF and the FRAIL, indicating that malnutrition affects frailty through an indirect path via sarcopenia. Furthermore, the PA level moderates this mediator effect, with sarcopenia serving as a mediator in older adults with moderate a PA level but not in those with a low PA level. These findings reveal that it may be beneficial to consider PA level in combination with malnutrition and sarcopenia in the management and prevention of frailty in community-dwelling older adults.

**Supplementary Information:**

The online version contains supplementary material available at 10.1186/s11556-024-00354-7.

## Introduction

The global population is aging rapidly, and the number of people aged ≥ 65 years, a critical population for healthcare providers, is steadily increasing [1]. In 2022, about 10% of the global population was aged ≥ 65 years, and it is estimated to increase to almost 17% by 2050 [2]. Similar trends are expected to occur in Turkey, where people aged ≥ 65 years are expected to rise from nearly 9% in 2022 to around 21% in 2050 [2]. This is likely to increase the prevalence of age-related conditions such as malnutrition, sarcopenia, and frailty, which are common and overlapping conditions in older adults [3].

Frailty is a multi-system dysregulation characterized by increased vulnerability, decreased physiological reserves, and reduced ability to respond to external stressors [4]. Malnutrition is characterized by inadequate intake or uptake of protein and energy, leading to altered body composition and poor clinical outcomes [5]. Finally, sarcopenia is another age-related condition that is defined as a generalized skeletal muscle disorder characterized by progressive loss of muscle mass and function [6]. These age-related conditions, which are potentially reversible, are associated with multiple health-related negative outcomes including falls, fractures, disability, longer hospitalization duration, morbidity, and even mortality in older adults [7–9]. Although malnutrition, sarcopenia, and frailty are distinct, there are many similarities among the three conditions, and hence a substantial proportion of older adults are likely to have multiple or even all three conditions concurrently [10]. The few studies investigating the overlap of malnutrition, sarcopenia, and frailty in community-dwelling older adults have noted a prevalence of 7% and 13.6% [11, 12].

Although the association among malnutrition, sarcopenia, and frailty is not completely elucidated, it is widely accepted that malnutrition has a crucial role in the pathogenesis of both sarcopenia and frailty and is a substantial modifiable risk factor in the context of sarcopenia and frailty [13]. It has been emphasized in systematic reviews that nutritional status is significantly associated with sarcopenia [14] or frailty [15, 16]. Furthermore, it has been reported that malnutrition is associated with an approximately four- and five-fold higher risk of sarcopenia [17] and frailty [18], respectively.

To our knowledge, however, no study deals with these three conditions concurrently and examines the complex associations between them, especially the mediator role of sarcopenia in the association between malnutrition and frailty. Clarification of these associations will provide a reference point for understanding the complex associations of frailty and coping with frailty in older adults. Hence, the aims of the current study were to explore: (1) whether the risk of sarcopenia has a mediator effect on the association between risks of malnutrition and frailty; and (2) whether the physical activity (PA) level modulates this mediator effect in community-dwelling older adults. We hypothesized that the risk of sarcopenia has a mediator effect on the association between the risks of malnutrition and frailty and that the PA level moderates this mediator effect in community-dwelling older adults. These hypotheses are illustrated in Supplementary Figs. 1 and 2.

## Materials and methods

The study was accepted by the University of Health Sciences, Gülhane Scientific Research Ethics Committee (2023 − 151). All participants were informed about the study, and they signed a voluntary participation form that adhered to the protocols of the Helsinki Declaration before enrollment in the study. The inclusion criteria were; 1) aged ≥ 65 years and b) sufficient comprehension and speaking skills in Turkish. They were excluded if they were not community-dwellers and had neurological diseases. In this cross-sectional study, 593 community-dwelling older adults were reached using the method of snowball sampling from the province of Ankara, Turkey’s capital. Initially, 25 primary participants representing the target population of our study were identified from the researchers’ network. The primary participants were then asked to invite other individuals who might be eligible for the study. To obtain data, face-to-face interviews were conducted between May 2023 and Agust 2023 at the faculty of health sciences and a questionnaire including demographic characteristics, malnutrition risk, sarcopenic risk, frailty status, and physical activity (PA) level was administered by the researchers.

### Outcome measurements

The Mini Nutritional Assessment-Short Form (MNA-SF), which is recommended by the European Society for Clinical Nutrition and Metabolism to be used in older adults [19], was used for malnutrition screening. It is a validated 6-item instrument that provides a continuous score (0–14 points) and categorizes participants as having healthy nutritional status (12–14 points), at risk of malnutrition (8–11 points), or malnourished (0–7 points) [20]. The validity study of the MNA-SF in Turkish older adults was conducted by Sarıkaya et al. [21].

The SARC-F questionnaire, which addressed strength, assistance required for walking, standing up from a chair, climbing stairs, and falls, was used for sarcopenia screening. It is a validated 5-item tool that provides a continuous score (0–10 points), with a score of ≥ 4 indicating a risk of sarcopenia [22].

Frailty screening was executed using the 5-item FRAIL Scale, which measures fatigue, endurance, ambulation, illnesses, and loss of weight. Participants who scored 0 were regarded as robust, whereas those who scored 1–2 and ≥ 3 were regarded as pre-frail and frail, respectively [23]. Turkish validation of the FRAIL Scale was carried out by Hymabaccus et al. [24].

Physical activity (PA) level was assessed using the International Physical Activity Questionnaire Short Form (IPAQ-SF). It records the activity of four intensity levels, namely, vigorous-intensity, moderate-intensity, walking, and sitting [25]. When determining the total score, the duration and frequency of various activities were noted. Activities that lasted at least 10 min at a time were considered. The duration, number of days of the activity per week, and metabolic equivalent (MET) values were multiplied to obtain the ‘MET-minute/week’ value. The duration in minutes of walking, moderate activity, and vigorous activity were multiplied by 3.3, 4, and 8 METs, respectively. According to the total MET value, individuals’ PA levels were classified as low if they were < 600 MET-minutes/week, moderate if they were between 600 and 3000 MET-minutes/week, and high if they were > 3000 MET-minutes/week [26].

### Statistical analysis

Data analyses were performed with IBM SPSS Statistics for Windows v26.0 (SPSS Inc., Chicago, IL, USA) and Hayes PROCESS macro v4.2. Before the statistical analysis was performed, the distribution of the data was examined using analytical (KS-SW tests) and visual methods (histogram and probability graphs). As the examinations revealed all quantitative variables were normally distributed, the Pearson correlation test was performed to evaluate bivariate associations [27].

To explore the complex associations among malnutrition, sarcopenia, and frailty, a moderation model and a moderated mediation model were generated with 5000 random sample bootstrapping confidence intervals using the Hayes PROCESS macro [28]. First, the mediator effect of sarcopenia on the association between malnutrition and frailty was tested using Model 4. Then, the moderator effect of PA level on this mediator effect was tested using Model 7. Malnutrition, frailty, sarcopenia, and PA level were regarded as the independent, dependent, mediator, and moderator variables, respectively. Both models were controlled for gender and age. A *p* value less than 0.05 was determined as an indicator of statistical significance.

## Results

The study comprised 593 community-dwelling older adults with a mean age of 71.35 ± 5.86 years, of whom 372 (62.73%) were female. The mean scores for MNA-SF, SARC-F, and FRAIL were 9.74 ± 2.31, 3.26 ± 2.57 and 1.53 ± 1.33, respectively. Malnourishment was present in 17.2%, risk of sarcopenia in 41.82%, and frailty in 26.64% of the participants. The majority of the participants had a low PA level (71.66%, *n* = 425/593), with a median of 99 (25th-75th percentile: 0-297) METs/week. Table 1 provides additional details about the participants’ demographic and clinical characteristics.

**Table 1:** Demographic and clinical characteristics of the participants

	All (*n* = 593)	Low PA level (*n* = 425)	Moderate PA level (*n* = 126)
**Age (years)**	71.35 ± 5.86	71.71 ± 6.07	70.07 ± 4.85
**Gender**			
Female	372 (62.73)	315 (68.03)	57 (43.85)
Male	221 (37.27)	148 (31.97)	73 (56.15)
**BMI (kg/m2)**	27.85 ± 4.82	27.97 ± 5.02	27.41 ± 3.98
**BMI classification**			
Underweight (< 18.5 kg/m2)	5 (0.84)	5 (1.08)	0 (0)
Healthy weight (18.5–24.99 kg/m2)	158 (26.64)	124 (26.78)	34 (26.15)
Overweight (≥ 25.00–29.99 kg/m2)	251 (42.33)	189 (40.82)	62 (47.69)
Obese (≥ 30.0 kg/m2)	179 (30.19)	145 (31.32)	34 (26.15)
**Presence of chronic disease (yes)**	502 (84.65)	404 (87.26)	98 (75.38)
**Educational status**			
Illiterate	93 (15.68)	77 (16.63)	16 (12.31)
Literate	80 (13.49)	63 (13.61)	17 (13.08)
Primary education	216 (36.42)	174 (37.58)	42 (32.31)
Secondary education	182 (30.69)	137 (29.59)	45 (34.61)
Tertiary education	22 (3.71)	12 (2.59)	10 (7.69)
**Physical activity (METs/week)**	198 (0-558)	99 (0-297)	990 (735–1386)
**MNA-SF (0–14 p)**	9.74 ± 2.31	9.61 ± 2.37	10.22 ± 2.03
Normal nutritional status (12–14 p)	228 (38.45)	166 (35.85)	62 (47.69)
At risk of malnutrition (8–11 p)	263 (44.35)	209 (45.14)	54 (41.54)
Malnourished (0–7 p)	102 (17.2)	88 (19.01)	14 (10.77)
**SARC-F (0–10 p)**	3.26 ± 2.57	3.65 ± 2.64	1.88 ± 1.73
No risk of sarcopenia (< 4 p)	345 (58.18)	240 (51.84)	105 (80.77)
Risk of sarcopenia (≥ 4 p)	248 (41.82)	223 (48.16)	25 (19.23)
**FRAIL (0–5 p)**	1.53 ± 1.33	1.70 ± 1.34	0.95 ± 1.09
Robust (0 p)	179 (30.19)	121 (26.13)	58 (44.62)
Pre-frailty (1–2 p)	256 (43.17)	197 (42.55)	59 (45.38)
Frailty (3–5 p).	158 (26.64)	145 (31.32)	13 (10)

Data are given as mean *±* standard deviation, number (percentage), or median (25th-75th percentile). SARC-F is a screening tool for sarcopenia; FRAIL is a screening tool for frailty; MNA-SF, Mini Nutritional Assessment-Short Form; PA, physical activity; BMI, body mass index

Table 2 displays the bivariate associations between the primary variables. Compared to those with a low PA level, participants with a moderate PA level had lower SARC-F and FRAIL scores, but higher MNA-SF scores (*r*=-0.285, *p* < 0.001; *r*=-0.235, *p* < 0.001; *r* = 0.109, *p* < 0.001, respectively). Higher MNA-SF scores were associated with lower SARC-F and FRAIL scores (*r*=-0.339, *p* < 0.001; *r*=-0.326, *p* < 0.001). There was also a significant association between SARC-F and FRAIL scores (*r* = 0.691, *p* < 0.001).

**Table 2:** The correlation matrix of the primary variables

	MNA-SF	SARCF	FRAIL	PA level
MNA-SF	1			
SARCF	-0.339^*^	1		
FRAIL	-0.326^*^	0.691^*^	1	
PA level	0.109^*^	-0.285^*^	-0.235^*^	1

Pearson Correlation Test. *, *p* value < 0.01; SARC-F is a screening tool for sarcopenia; FRAIL is a screening tool for frailty; MNA-SF, Mini Nutritional Assessment-Short Form; PA, physical activity; PA level is coded as low (0) and moderate (1)

**Fig. 1:**
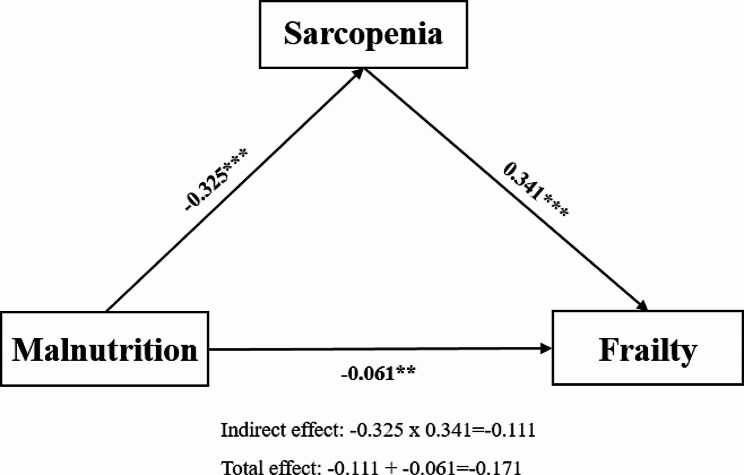
The mediation model illustrates the mediator role of sarcopenia in the association between malnutrition and frailty. Notes: Unstandardized regression weights are shown for the associations between each variable. *p < 0.05, **p < 0.01, ***p < 0.001

In accordance with a mediation model, the MNA-SF had a significant effect on the SARC-F (B=-0.325; *p* < 0.001) and the SARC-F, in turn, had a significant effect on the FRAIL (B = 0.341; *p* < 0.001). As a next step, the total effects, direct effects (independent of SARC-F), and indirect effects (mediated by SARC-F) of the MNA-SF on the FRAIL were examined. The total (B=-0.171; *p* < 0.001), direct (B=-0.061; *p* = 0.001), and indirect (B=-0.111; bootstrap CI did not include zero, which indicates a significant effect) effects were significant, showing that 65% of the association between the MNA-SF and the FRAIL was explained by the SARC-F acting as a mediator (Table 3; Fig. 1).

**Table 3:** The mediating effect of SARC-F in the association of the MNA-SF and FRAIL

Model pathways		B	95% CI	t	*p*
MNA-SF→SARC-F		-0.325	-0,404 to -0.245	-8.037	**< 0.001**
SARC-F→FRAIL		0.341	0.307 to 0.376	19.447	**< 0.001**
MNA-SF→SARC-F→FRAIL					
	Direct effects	-0.061	-0.096 to -0.025	-3.356	**0.001**
	Indirect effects	-0.111	-0.142 to -0.080	-	**-**
	Total effects	-0.171	-0.215 to -0.128	-7.788	**< 0.001**

% Total effects mediated by MNA-SF: **65%**

The model is adjusted for gender and age. Bootstrap *N* = 5000. B: unstandardized coefficients; CI: confidence interval; SARC-F is a screening tool for sarcopenia; FRAIL is a screening tool for frailty; MNA-SF, Mini Nutritional Assessment-Short Form

**Fig. 2:**
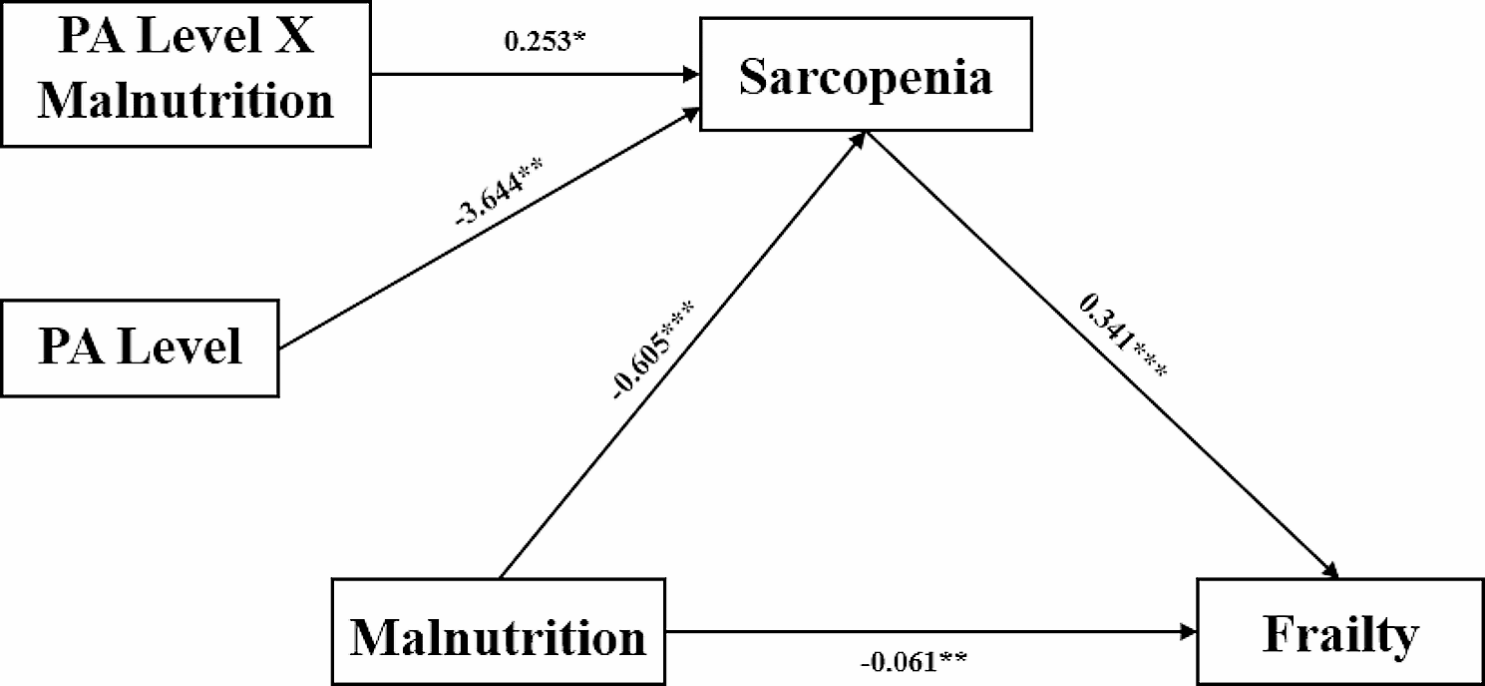
The moderated mediation model illustrates the moderator role of physical activity level and the mediator role of sarcopenia in the association between malnutrition and frailty. Notes: Unstandardized regression weights are shown for the associations between each variable. *p < 0.05, **p < 0.01, ***p < 0.001

The results of the moderated mediation analysis are given in Fig. 2; Table 4. The analysis indicated that the MNA (B=-0.605; *p* < 0.001) and the PA level (B=-3,644; *p* = 0.001) had significant negative effects on the SARC-F. The interaction effect of the MNA and the PA level (B = 0.253; *p* = 0.016) was significant, demonstrating that the association between the MNA and the SARC-F was moderated by the PA level. Furthermore, the MNA (B=-0,061; *p* = 0.001) and the SARC-F (B = 0,341, *p* < 0.01) had negative and positive significant effects on the FRAIL, respectively.

**Table 4:** Moderated mediation model with SARC-F as a mediator and PA level as a moderator

Model pathways	B	95% CI	t	*p*
MNA-SF→SARC-F	-0.605	-0.859 to -0.350	-4.672	**< 0.001**
PA→SARC-F	-3.644	-5.769 to -1.519	-3.368	**0.001**
MNA-SF*PA→SARC-F	0.253	0.047 to 0.460	2.407	**0.016**
MNA-SF→FRAIL	-0.061	-0.096 to -0.025	-3.356	**0.001**
SARC-F→FRAIL	0.341	0.307 to 0.376	19.447	**< 0.001**

The model is adjusted for gender and age. Bootstrap *N* = 5000. B: unstandardized coefficients; CI: confidence interval; SARC-F is a screening tool for sarcopenia; FRAIL is a screening tool for frailty; MNA-SF, Mini Nutritional Assessment-Short Form

**Fig. 3:**
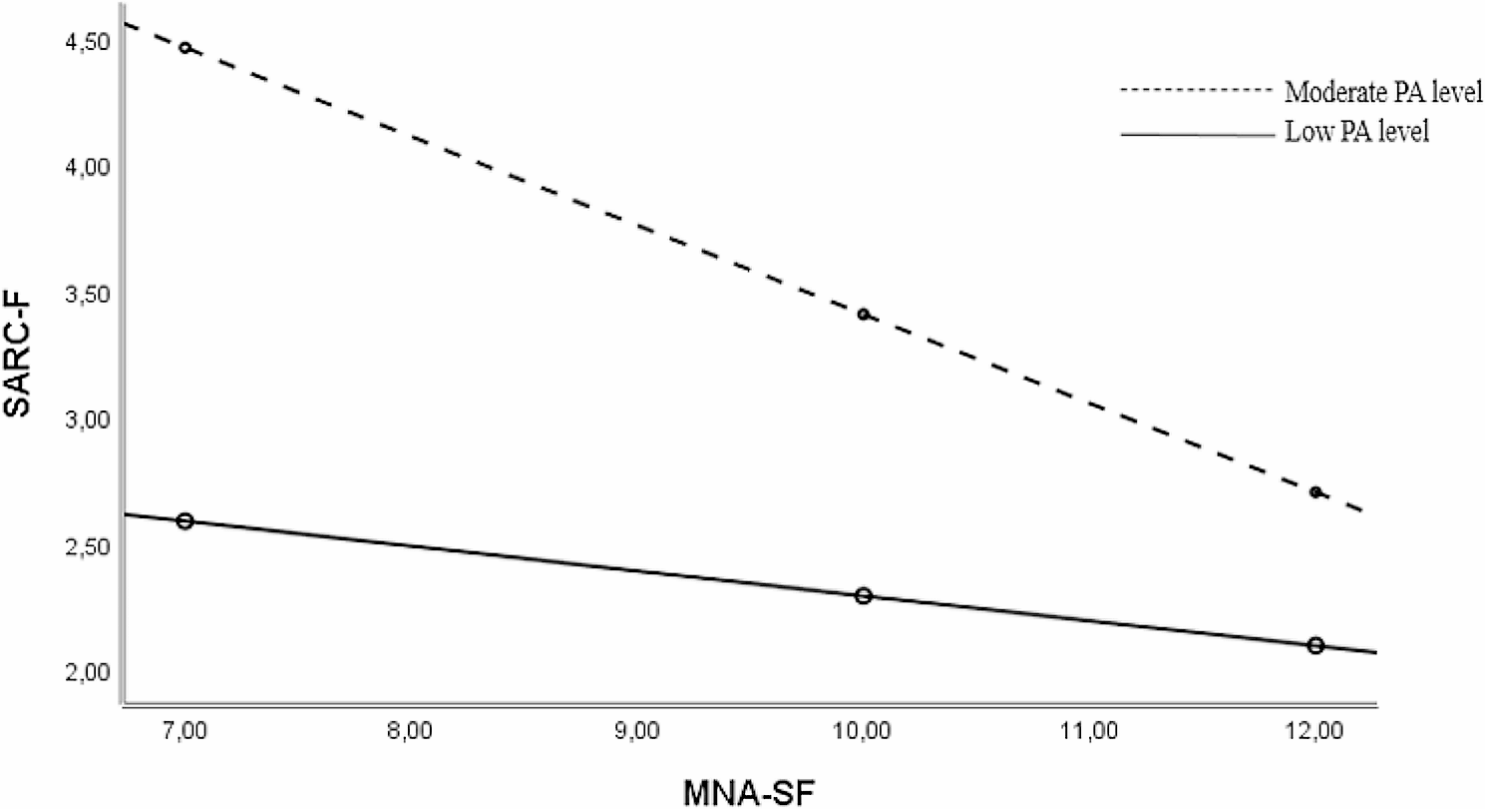
The conditional effect of MNA-SF on SARC-F. SARC-F is a screening tool for sarcopenia; MNA-SF, Mini Nutritional Assessment-Short Form

The MNA had a significant indirect effect on the FRAIL in participants with a moderate PA level (B=-0.120). However, in participants with a low PA level, the MNA (B=-0.034, bootstrap CI included zero, which indicates no significant effect) had no significant indirect effect on the FRAIL (Table 5; Fig. 3). These findings demonstrated that the indirect effect of the MNA on the FRAIL was relatively increased in the moderated mediation model (B=-0.120), in which there was a moderate PA level compared to the mediation model (Table 3: B=-0.111).

**Table 5:** Conditional indirect effects of the MNA-SF on FRAIL at values of the PA level

PA level	B	95% CI
Moderate	-0.120	-0.154 to -0.085
Low	-0.034	-0.078 to 0.009

B: unstandardized coefficients; CI: confidence interval. Moderated mediation index = 0.086 (0.03 to 0.14)

## Discussion

To our knowledge, there exists no study in the literature dealing with the risks of malnutrition, sarcopenia, and frailty concurrently and examining the associations between them. Thus, the current study is the first to show that the risk of sarcopenia has a mediator effect on the association between the risks of malnutrition and frailty, accounting for the majority (65%) of the association, and that PA level modulates this mediator effect in community-dwelling older adults. Furthermore, the risk of sarcopenia did not mediate the association between the risks of malnutrition and frailty in older adults with a low PA level; however, the risk of sarcopenia functioned as a mediator and relatively increased the association between the risks of malnutrition and frailty in older adults with a moderate PA level.

### Association of the risk of malnutrition with the risks of frailty and sarcopenia

Malnutrition, resulting in a deterioration in overall health, is prevalent in older adults and its prevalence generally increases with aging [29]. It is often associated with inflammatory catabolism and leads to body composition changes and loss of muscle mass and strength, implying that malnutrition is an essential contributor to the pathogenesis of both sarcopenia and frailty [30, 31]. In a systematic review, Bloom et al. declared that sarcopenia was associated with diet quality [14]. Lorenzo-López et al. reported in a systematic review that frailty was associated with low intake of several micro- and macronutrients, diet quality, and antioxidant capacity [15]. These studies implied that malnutrition was associated with both sarcopenia and frailty in older adults, which is comparable to the current study.

### Association between the risks of sarcopenia and frailty

Sarcopenia and frailty have received considerable attention and have been addressed concurrently in recent geriatric research as both are common in older adults, are associated with health-related negative outcomes, and have the potential to be reversible [7]. They are related geriatric syndromes with similar clinical manifestations, including muscle weakness, slow walking speed, and reduced physical activity, and similar pathophysiologic pathways, including dysregulation of metabolic, musculoskeletal, and hormonal pathways [32]. Yet, the nature of the association between sarcopenia and frailty, which eventually overlap because of their close association with the physiological and pathological aging process, remains unclear [33]. In the current study, we addressed a hypothesis that sarcopenia is associated with frailty in community-dwelling older adults rather than addressing the direction and underlying mechanism of the association. Sarcopenia was associated with frailty in the current study, which is similar to previous studies reporting a positive association between sarcopenia and frailty in older adults [34, 35]. Furthermore, the current study is the first to deal with the mediator role of sarcopenia in the association of frailty with malnutrition. Our findings provided a novel contribution that this association in older adults is partially mediated by sarcopenia, highlighting how malnutrition affects frailty indirectly through sarcopenia. These findings shed light on the complex associations of frailty and provide a reference point for coping with frailty in older adults.

### Association between physical activity and the risk of sarcopenia

It has been clearly documented in several systematic reviews [36–38] and studies [39–41] that PA and sarcopenia are significantly associated and PA is an effective prevention and treatment strategy for sarcopenia in older adults. Although evidence on different dimensions of PA such as type, intensity, frequency, and duration is limited, it has been reported that PA can prevent, delay, or even treat sarcopenia by increasing muscle mass, strength, and physical performance in older adults [36, 38].

The current study had several limitations. First, snowball sampling was used to include older adults from Ankara province, which may weaken the generalizability of the findings and the representativeness of the sample. Future studies should use random sampling methods that provide members of the target population with an equal possibility of being chosen for sampling. Second, due to the design of our study, only cross-sectional associations could be examined, which is insufficient to define the causal direction of the association between variables. Nevertheless, our findings may provide preliminary data and shed light on further longitudinal or experimental studies. Third, the IPAQ, a self-reported questionnaire, was employed to assess physical activity. Objective measures of physical activity, such as accelerometry, should be used in future studies to obtain reliable data. Fourth, participants in this study had a low/moderate PA level. Studies including participants with a high PA level are needed.

## Conclusions

The SARC-F mediates the association between the MNA-SF and the FRAIL, indicating that the risk of malnutrition affects the risk of frailty through an indirect path via the risk of sarcopenia. Furthermore, PA level moderates this mediator effect, with the risk of sarcopenia serving as the mediator in older adults with a moderate PA level but not in those with a low PA level. Thus, to effectively prevent or manage frailty in older adults, physical activity should be taken into consideration as well as the risks of malnutrition and sarcopenia.

### Electronic supplementary material

Below is the link to the electronic supplementary material.

Supplementary Material 1

## Data Availability

The data that support the findings of this study are available from the corresponding author upon reasonable request.
