# Supplementary material for: Exploring the complex associations among risks of malnutrition, sarcopenia, and frailty in community-dwelling older adults
Source: Eur Rev Aging Phys Act. 2024 Jul 9;21:18. doi: 10.1186/s11556-024-00354-7 (PMC11232342; doi:10.1186/s11556-024-00354-7)
Supplement: Supplementary file 1 — Supplementary Material 1 [file 11556_2024_354_MOESM1_ESM.docx]

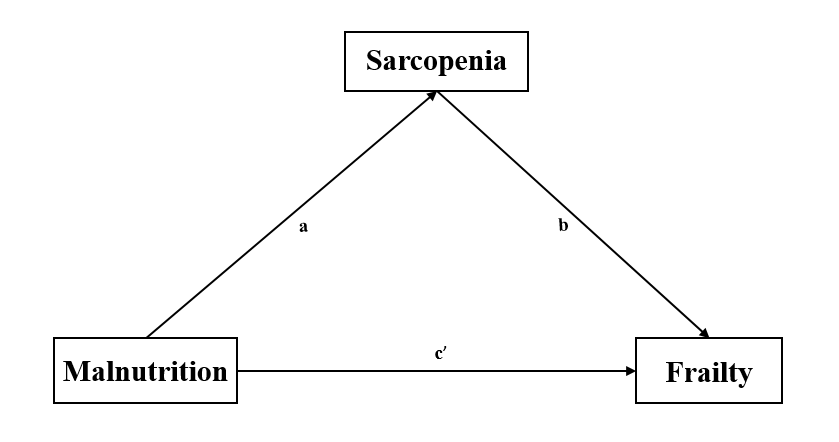


**Supplementary Figure 1:** Hypothesized mediation model. The direct effects of malnutrition on frailty, independent of sarcopenia, are shown with c’. Indirect effects include (a) the association between malnutrition and sarcopenia and (b) the association between sarcopenia and frailty. Total effects would be the sum of all of these, indicating the overall association between malnutrition and frailty.


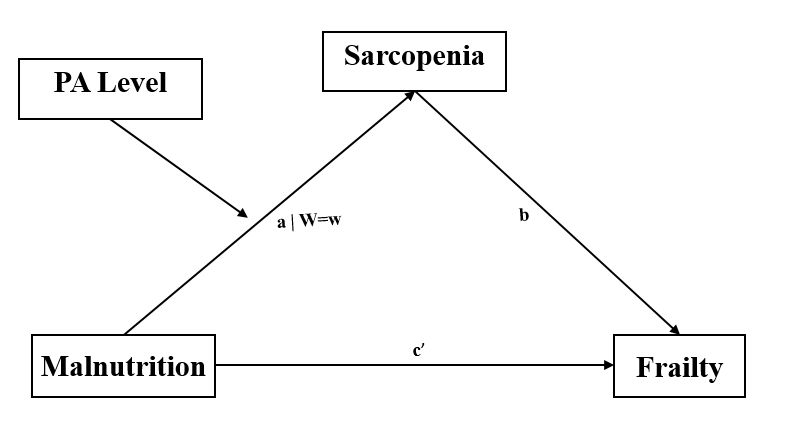


**Supplementary Figure 2.** Hypothesized moderated mediation model. The direct effects of malnutrition on frailty, independent of sarcopenia, are shown with c’. Indirect effects include (a|W=w) the association between malnutrition and sarcopenia depending on the value of W (the moderator) and (b) the association between sarcopenia and frailty.
